# Effects of Fetal Bovine Serum on Biofilm Formation and Cell Attachment of *Candida glabrata*

**DOI:** 10.3390/microorganisms14102125

**Published:** 2026-09-22

**Authors:** Barbara E. Vecci, Sarang N. Deshpande, Jonathan Baudin, Katy C. Kao

**Affiliations:** Department of Chemical and Materials Engineering, San Jose State University, San Jose, CA 95192, USA

**Keywords:** *Candida glabrata*, surface coating, HI FBS, fungal biofilm, cell attachment

## Abstract

*Candida glabrata* (*Nakaseomyces glabratus*) is an opportunistic human fungal pathogen. *Candida* spp., including *C. glabrata*, can adhere to surfaces, including the human epithelial mucosa and medical devices, to form biofilms, which can lead to high mortality rates. Heat-inactivated fetal bovine serum (HI FBS) is commonly used as a component in plate-based fungal biofilm experiments as a readily available serum model. However, the effect of HI FBS on *C. glabrata* biofilm formation in in vitro biofilm assays has not been systematically investigated. The results of crystal violet staining for biomass measurements, XTT reduction for metabolic activity quantification, confocal microscopy of biofilms and growth kinetic measurements showed a negative influence of the HI FBS on cell attachment but not growth rates, which resulted in reduced biofilm formation. These findings identify serum exposure as an important experimental variable in *C. glabrata* biofilm assays that can potentially disrupt the interaction of cells and abiotic surfaces such as those used in medical device applications.

## 1. Introduction

Infections by *Candida glabrata* (now called *Nakaseomyces glabratus*) account for 10–30% of candidemia cases [1]. Biofilms are implicated in ~70% of microbial infections and play a major role in nosocomial infections [2]. High mortality rates are associated with fungal biofilms, especially in immunocompromised patients, due to bloodstream infections caused by *Candida* species [3,4]. An in vivo investigation of catheters collected from intensive care unit patients revealed that 56.4% developed biofilms, with *C. albicans* being the most common biofilm-forming species, followed sequentially by *C. glabrata*, *C. parapsilosis*, and *C. tropicalis* [5]. Unlike *C. albicans*, *C. glabrata* does not form hyphae. *C. glabrata* biofilms consist of yeast cells either as compact layers or in smaller clusters [6,7]. Biofilms are communities of microorganisms within an extracellular matrix (ECM) that are attached to biotic or abiotic surfaces [3]. The extracellular matrix protects against host defenses and antifungal agents [8]. Microbial biofilms, including those involving *C. glabrata*, can form on medical devices, posing significant clinical challenges [3,9].

The biofilm formation process can be affected by several factors, including interactions among yeast cells, host-derived factors, and surface properties. Cell adhesion to biotic or abiotic surfaces is the first step in the biofilm formation process, followed by cell proliferation on the surface, formation and maturation of the biofilm, and dispersion of the fungal cells from the matrix to the environment to initiate adhesion at a secondary site [3,10]. Cell attachment involves physicochemical properties that are specific (adhesins) or non-specific (hydrophobic and electrostatic binding forces) [9,11]. Studies have shown that *Candida* species can adhere to multiple surfaces such as polystyrene, polypropylene, silicone, different types of resins, polyvinyl chloride, and glass [12].

In vitro biofilm experiments are often performed by pre-treating the surface with serum such as fetal bovine serum, which can stimulate cell growth due to its composition of essential substances for cell proliferation and maintenance enhancing growth factors [13,14]. Host proteins like fibronectin (present in FBS) can be adsorbed onto medical device surfaces [15]. Silicone surfaces coated with human serum have been found to stimulate *C. albicans* biofilm formation [16,17]. Serum coating also led to increased *C. glabrata* biofilm formation on acrylic surfaces as the serum concentration increased [18]. However, conflicting results have also been reported, showing that serum inhibits biofilm formation in *C. albicans* [17,19]. *C. glabrata* may respond differently from *C. albicans* to the presence of serum and serum components due to differences in cell morphology, adhesin repertoires, cell wall organization, and biofilm architecture. In this work, we investigated the effects of serum (using fetal bovine serum as a model) on *C. glabrata* planktonic growth kinetics and biofilm formation on surfaces commonly used in in vitro biofilm assays using crystal violet staining, XTT reduction assays and confocal microscopy.

## 2. Materials and Methods

### 2.1. Strain and Growth Conditions

A *Candida glabrata* (ATCC 2001) strain marked with yellow fluorescent protein (YFP) was used in this study [20]. Prior to biofilm growth, strains were cultured overnight in 10 mL of YPD (Yeast Peptone Dextrose) medium at 37 °C and 230 rpm. For biofilm growth, cells were cultured in RPMI 1640 (Gibco, Waltham, MA, USA) supplemented with 34.53 g/L MOPS (3-(N-morpholino) propane sulfonic acid, Sigma, St. Louis, MO, USA) and titrated to pH 7.0 using 10 N NaOH.

### 2.2. Preparation of Surfaces with HI-FBS

Heat-inactivated fetal bovine serum (HI FBS, Corning, Corning, NY, USA, catalog number 35011CV) was used to coat glass and vinyl coverslips or wells in 96-well microtiter plates [21,22]. These surfaces were selected because polystyrene microtiter plates are widely used for high-throughput biofilm quantifications (including characterizing clinical isolates), and optically clear glass and vinyl coverslips allow imaging studies and comparisons of substrates with different surface properties. Previous work showed more abundant biofilm formation when surfaces were preincubated with FBS [22]. For 96-well plates, 100 μL of HI FBS was added to designated wells. Glass and plastic coverslips were pre-sterilized with 70% alcohol and autoclaved, then placed in Petri dishes containing 10 mL of HI FBS. Coverslips and 96-well plates were incubated with HI FBS at 37 °C for 24 h unless otherwise specified and aspirated prior to use. The effects of heat inactivation on FBS (Corning, catalog number 35010CV) were also assessed by incubating normalized cells for biofilm formation in wells pre-treated with no FBS, in-house heat-inactivated FBS (at 56 °C for 30 min), and non-heat-inactivated FBS, from the same batch.

### 2.3. Biofilm Formation

Overnight cultures were centrifuged at 2700 rpm for 5 min and washed once with 1× PBS (phosphate-buffered saline). The cell pellet was resuspended in 5 mL of RPMI 1640. The cell concentration was measured using a cell counter (Cellometer Spectrum, Revvity, Waltham, MA, USA), and the cell suspension was normalized to a final concentration of 1 × 10^7^ cells/mL with RPMI 1640 medium supplemented with 34.53 g/L MOPS and titrated to a pH 7.0 using 10 N NaOH.

For 96-well assays, 100 μL of the normalized culture was added to each well. To prevent evaporation, the wells along the edges of the plate were filled with 100 µL of RPMI 1640 and not used for measurements. The plates were incubated at 37 °C for either 90 min or 24 h to assess the effects of HI FBS on initial cell attachment or biofilm growth, respectively.

To evaluate the effect of supplementing RPMI 1640 with HI FBS (2%, 5% and 10% by volume) on biofilm formation, normalized cells were incubated in HI FBS-supplemented RPMI 1640 medium for 24 h at 37 °C in non-pre-coated wells.

After incubation, the plates were rinsed with 100 μL of 1× PBS to remove any planktonic (non-adherent) cells, followed by quantification using either a crystal violet staining (Fisher Chemical, Pittsburgh, PA, USA) or 2,3-Bis-(2-Methoxy-4-Nitro-5-Sulfophenyl)-2H-Tetrazolium-5-Carboxanilide Tetrazolium Salt (XTT, Sigma) assay.

For biofilm formation on coverslips (plastic or glass), 9 sterile coverslips were placed in a sterile Petri dish. A 20 mL volume of normalized cell suspension (1 × 10^7^ cells/mL) in RPMI 1640 medium supplemented with 34.53 g/L MOPS and titrated to pH 7.0 was added to each Petri dish and incubated at 37 °C for either 90 min or 24 h to assess the effects of HI FBS on initial cell attachment or biofilm growth, respectively. After the incubation, the coverslips were transferred to 6-well plates (one per well) for crystal violet or XTT assays.

### 2.4. Crystal Violet Assay

For 96-well plates, after the biofilms were rinsed with PBS, excess PBS was removed, and 100 μL of absolute methanol was added to each well for cell fixation. After 15 min, the methanol was removed and allowed to dry completely for 15 min. A 100 μL volume of 0.1% (*w*/*v*) crystal violet solution was added to each well and stained for 15 min. After staining, the excess crystal violet in each well was removed. The plate was then rinsed with DI water to remove any unbound dye, and the plate was allowed to air dry. Then, 130 μL of 33% (*v*/*v*) acetic acid was added to each well to recover the bound crystal violet for quantification. Quantities of 100 μL of each sample and 100 µL of DI water were transferred into a black-walled, clear-bottom 96-well plate for absorbance measurements using a plate reader (BioTek Epoch 2 Microplate Reader, Agilent Technologies, Santa Clara, CA, USA) at 590 nm.

The biofilm-cultured coverslips were washed with 1 mL of PBS in each well in a 6-well plate, and then the excess was removed. A 1 mL volume of methanol was added to each well and incubated for 15 min for cell fixation. Excess methanol was then removed and allowed to dry completely for 15 min. For staining, 1 mL of crystal violet was added to each well and incubated for 15 min. Excess crystal violet was then removed and rinsed with DI water to remove any unbound dye. Each plate was left open for 15 min to dry before 0.5 mL of 33% acetic acid was added to each well to recover the bound crystal violet for quantification. Quantities of 100 μL of each sample + 100 µL of DI water were transferred into a black-walled clear-bottom 96-well plate for absorbance measurements in a plate reader (BioTek Epoch 2 Microplate Reader) at 590 nm. For data analysis, all technical replicates were averaged for each biological replicate, resulting in one value per biological replicate. Crystal violet staining data was analyzed using the 2-tailed Student *t*-test with unequal variance in Excel as the effects of FBS on biofilm formation may be negative or positive.

### 2.5. XTT Assay

XTT assay reagents were freshly prepared by mixing XTT and menadione before each use as described previously with minor modifications [7]. Briefly, XTT (Sigma Aldrich, St. Louis, MO, USA) was dissolved in PBS to a final concentration of 0.5 mg/mL, filter-sterilized, divided into single-use aliquots and stored at −80 °C. Menadione (Sigma Aldrich) was dissolved in 100% acetone to a final concentration of 10 mM, divided into single-use aliquots and stored at −80 °C. Prior to use, aliquots of menadione (used as an electron-coupling agent) and XTT were thawed at room temperature and mixed at a 1:10 menadione:XTT (*v*/*v*) ratio for a final concentration of 1 µM menadione. A 100 µL volume of the XTT/menadione solution was added to each well. To reduce evaporation during the experiment, PBS was added to the empty wells along the edges of the 96-well plates. The plate was covered with aluminum foil and incubated for 3 h at 37 °C in the dark. After incubation, 80 µL of the supernatant was transferred to a black-walled, clear bottom 96-well plate, and the absorbance at 490 nm was measured using a microplate reader (BioTek Epoch 2 Microplate Reader). For data analysis, all technical replicates were averaged for each biological replicate, resulting in one value per biological replicate. XTT data was analyzed using Excel with the unpaired two-tailed Student *t*-test. Note that the XTT assay was used as a measure of relative metabolic activity rather than for quantification of viable cell concentrations in this study.

### 2.6. Planktonic Growth Assay

Planktonic growth kinetics were evaluated in RPMI 1640 supplemented with different concentrations of HI FBS (2%, 5% and 10%). The overnight cell culture was normalized to a final concentration of 1 × 10^7^ cells/mL in supplemented RPMI 1640 and cultured for 24 h at 37 °C in 96-well plates in a plate reader (BioTek Epoch 2 Microplate Reader) with shaking. Growth was monitored at 600 nm every 20 min. The growth kinetics data was analyzed using the quantitative growth curve evaluation (QurvE v1.1) [23] for growth rate (μ) and lag time (λ) values. For data analysis, all technical replicates were averaged for each biological replicate, resulting in one value per biological replicate. Statistical comparisons were performed for FBS supplementation versus no FBS supplementation using the unpaired two-tailed Student *t*-test in Excel.

### 2.7. Confocal Microscopy Imaging

For confocal microscopy imaging, biofilms were formed on glass coverslips as described above. Coverslips with biofilms were removed from their Petri dishes and transferred to a six-well plate. Each coverslip was rinsed with 1 mL of PBS to remove planktonic cells, and excess PBS was removed using vacuum aspiration. A 1 mL volume of 4% (*v*/*v*) formaldehyde solution was added to each well for cell fixation. After 30 min, the formaldehyde was removed and the sample was allowed to dry completely for 15 min. The plates were rinsed with 1 mL of PBS three times to remove all residual formaldehyde. Calcofluor White solution was prepared by diluting a stock solution (Sigma Aldrich) to a 1:10 dilution in phosphate-buffered saline (PBS). Then 1 mL of Calcofluor White solution in PBS and 1 mL of 10% potassium hydroxide were added to each well, and the mixture was stained for 15 min. The excess dye solution was aspirated after the staining. The plate was rinsed with PBS, and the excess was removed via vacuum aspiration. The plates were left open to completely dry inside a biosafety cabinet for about 15 min.

To facilitate imaging, coverslips were placed on glass microscope slides to establish a uniform imaging chamber. A thin layer of petroleum jelly was applied to the surface of the spacers to secure them to the coverslip and prevent air bubbles that could compromise image quality. The spacers maintained a consistent 1.5 mm gap between the slide and coverslip, allowing optimal focusing while preventing direct contact with the specimen. Since imaging was performed using an upright confocal microscope, the coverslip was carefully inverted, with the sample side facing downward. Coverslips were handled exclusively with precision tweezers throughout the preparation process to avoid contamination or damage.

Images were acquired using a Zeiss LSM 700 Upright Confocal Microscope (Carl Zeiss Microscopy GmbH, Oberkochen, Germany) with 20× and 40× objectives. Zeiss ZEN 2011 SP7 software was used for image acquisition and subsequent processing. Excitation of the YFP expressed by the *C. glabrata* strain was achieved using a 488 nm laser. Imaging was performed in frame scan mode at resolutions of 2048 × 2048 and 512 × 512 pixels.

Biofilm structure was analyzed from confocal z-stacks using COMSTAT2 v2.1 in ImageJ (1.48v) [24,25]. Using the Bio-Formats plugin, the z-stacks were transformed from .czi to OME-TIFF format and analyzed independently. All stacks under both conditions were subjected to a consistent manual intensity threshold of 50 (on an 8-bit, 0–255 scale). Because automated Otsu thresholding produced T = 1 for these low-background fluorescence images, thresholds were manually selected according to the original COMSTAT protocol [24]. The biofilm-positive signals ranged from roughly 80 to 250. Before quantitative analysis, the threshold T = 50 was visually identified from representative intensity histograms to lie between background autofluorescence and biofilm signals.

To restrict the measurements to biomass anchored to the substratum, COMSTAT2 was run with connected volume filtering (CVF) enabled, slice smacking set to 2, and a minimum colony size of 100 pixels. The biovolume (µm^3^/µm^2^), substratum coverage (%), roughness coefficient Ra*, mean thickness throughout the field and in biomass-positive areas (µm), maximum thickness (µm), and surface-to-volume ratio (µm^−1^) were all measured for each stack.

The confocal imaging was carried out across three separate biological replicates (independent biofilm samples generated on different days), with several samples per day and imaging locations per sample. Per-position measurements were averaged within samples, and per-sample means were averaged per biological replicate, resulting in one mean value per biological replicate (3 biological replicates total). Unpaired two-tailed Student *t*-tests were used to compare the average values of different parameters between HI FBS and no-HI FBS samples. Values are provided as the mean +/− standard deviation of three biological replicates.

## 3. Results and Discussion

### 3.1. HI FBS Reduces C. glabrata Biofilm Formation on Different Materials

To explore whether HI FBS coating influences *C. glabrata* biofilm formation, wells in 96-well plates (polystyrene) were either pre-coated with HI FBS or not coated prior to the addition of cells for biofilm growth. The amounts of biofilm formed in 96-well plates with or without HI FBS coating were quantified using crystal violet staining after 24 h. As shown in Figure 1A, the amount of biofilm formed decreased significantly (~83%) in the presence of HI FBS coating when compared to controls without HI FBS coating.

Since biofilm formation can be influenced by different surface properties [26], the effects of HI FBS on biofilm formation on vinyl and glass surfaces were also explored. In the absence of HI FBS, *C. glabrata* biofilm formation was more than 70% higher on vinyl compared to glass (Figure 1B,C), after differences in the total surface areas of the coverslips were considered. Reduced hydrophobicity has been found to negatively affect cell adhesion and biofilm formation in yeast [27,28]. Glass surfaces tend to be more hydrophilic than plastic in general, which likely explains the reduction in biofilm formation observed on glass coverslips. When the surfaces were coated with HI FBS, reductions in biofilm formation were observed on both vinyl and glass compared to uncoated surfaces, with a larger effect observed on glass (~66% vs. ~48% reduction on glass and vinyl, respectively). It is important to note that even though a 66% reduction by HI FBS was observed on glass, the difference was not statistically significant (*p*-value 0.07 using a two-tailed *t*-test) due to having only three biological replicates. However, the biofilm reduction trend was consistent across all surfaces tested.

### 3.2. Reduction in Biofilm Formation by FBS Is Independent of Heat Inactivation or Duration of Coating

Heat inactivation of FBS is routinely used to inactivate components of serum that may inhibit or interfere with cell culture experiments. To evaluate whether any serum component(s) that can affect biofilm formation may have been generated due to the heat inactivation process of FBS, non-heat-inactivated and heat-inactivated FBS from the same batch were compared for their ability to influence *C. glabrata* biofilm formation using crystal violet staining. As shown in Figure 2, heat inactivation had no effect on biofilm formation by *C. glabrata*, indicating that the anti-biofilm effect of serum is largely retained following heat treatment. This result suggests that components present in FBS that influence biofilm formation are neither generated nor inactivated by heat treatment under the conditions tested.

A prior study also found that heat-inactivated serum retained biofilm inhibitory effects in *Staphylococcus aureus* and that the inhibitor(s) is likely not proteinaceous [29]. Whether the same serum components that influenced *Staphyloccocus aereus* and *C. glabrata* biofilms are the same is unknown and cannot be inferred from the data. Since heat inactivation of FBS had minimal effects on fungal biofilm formation, HI FBS was used for all other experiments.

In addition, impacts of the duration of well coating (5 min, 2 h, and 24 h) on the effects of HI FBS on biofilm formation were also assessed to determine whether adsorption kinetics may play a role. As shown in Supplementary Figure S1, no significant differences in biofilm formation were observed in wells coated with HI FBS for 5 min, 2 h or 24 h.

### 3.3. Effects of HI FBS on C. glabrata Biofilm Metabolic Activity

To assess whether the negative effects of HI FBS on *C. glabrata* biofilm formation observed using crystal violet staining were correlated with cell metabolic activity, an XTT reduction assay was performed. This colorimetric assay measures the metabolic activity of viable cells through the reduction of the tetrazolium salt XTT to a water-soluble orange formazan product, facilitated by cellular mitochondrial dehydrogenase activity. Thus, XTT reduction measurements are a measure of relative metabolic activity rather than a direct measure of the viable cell concentration. Biofilms in 96-well plates were incubated for 24 h under two conditions, with and without HI FBS pre-coating, then assessed for biofilm metabolic activity.

The results in Figure 3 show that wells pre-coated with HI FBS exhibited a decrease (although not statistically significant, *p*-value 0.19) in XTT reduction compared to those without the pre-coating step, suggesting that the reduction in biofilm biomass measured via crystal violet may be correlated with a reduction in relative metabolic activities of cells in the biofilms. However, the observed effects of HI FBS from XTT reduction were significantly weaker than those observed from crystal violet staining (reduction of 24% using the XTT assay versus 83% using crystal violet). Differences in overall biofilm formation measured by crystal violet staining and cellular metabolic activity measured using XTT have been previously observed [30]. Since crystal violet binds to negatively charged molecules on cells and within the biofilm matrix, the results suggest that while HI FBS significantly affected overall biofilm formation (including extracellular polymeric substances and attached cells), the effects on metabolic activity appeared to differ. Since the viable cell concentrations in the biofilms were not quantified, the possibility of greater XTT-reducing activity per attached cell remains unresolved.

### 3.4. HI FBS Reduces the Initial Attachment of C. glabrata

To evaluate the effect of HI FBS on the initial adhesion of *C. glabrata* to abiotic surfaces, cells were incubated for 90 min at 37 °C in 96-well plates with or without HI FBS coating. After the incubation period, non-adherent cells were gently removed by washing the plates with PBS. The metabolic activity of adherent cells was assessed using the XTT reduction assay. A 34% decrease (although not statically significant; *p*-value = 0.08) in XTT reduction was observed in the presence of HI FBS compared to uncoated controls (Figure 4A), suggesting that HI FBS may reduce initial attachment of *C. glabrata* to the surface.

The total cell biomass was also quantified using crystal violet to assess potential changes in biomass after initial attachment. After cells were allowed to attach to HI FBS-coated or uncoated wells for 90 min at 37 °C, the total cell mass was quantified using crystal violet. As shown in Figure 4B, a significant reduction in stained biomass was observed in the HI FBS pre-coated condition compared to the uncoated control, confirming that HI FBS interfered with the initial adhesion of *C. glabrata* to the wells’ surfaces. Similar to the results observed in 24 h mature biofilms, a greater reduction in biofilm biomass formation was observed than in metabolic activity (51% with *p*-value < 0.001 vs. 34% and *p*-value = 0.08, respectively).

### 3.5. Medium Supplemented with HI FBS Reduces Biofilm Formation but Not Planktonic Cell Growth

The effects of supplementing RPMI 1640 with HI FBS instead of coating wells with HI FBS on biofilm formation were assessed by supplementing RPMI 1640 with 0%, 2%, 5%, or 10% (*v*/*v*) of HI FBS in non-pre-coated wells. After 24 h of incubation at 37 °C, the amount of biofilm was measured with crystal violet assay. The results showed that supplementing the medium with HI FBS led to a significant reduction in biofilm formation (Figure 5). And the effects of FBS did not appear to be dose-dependent within the 2–10% range tested. HI FBS supplementation also indicated that serum components can affect biofilm formation without prior surface conditioning, potentially through interactions with cells, adhesin regulation, or surface adsorption during incubation, although these mechanisms were not directly tested in this work.

RPMI 1640 supplemented with HI FBS (0%, 2%, 5%, and 10%) was also used to determine whether HI FBS affects planktonic cell growth. Growth kinetics were measured over a 24 h period at 37 °C, and the maximum specific growth rate and duration of lag time were determined using QurvE [23]. Interestingly, the presence of HI FBS did not significantly affect the maximum specific growth rate but reduced the duration of lag in a concentration-dependent manner (Table 1). Combined with the 90 min crystal violet biomass attachment data, these results are consistent with reduced initial cell attachment that contributed to lower 24 h biofilm biomass. However, surface proliferation may also have contributed to the overall reduction, which was not measured directly. The combination of surface pre-coating and continued exposure to serum, which may more closely model a medical device in a host, was not tested. It is unclear why increasing the concentration of FBS reduced the lag time in planktonic cultures. However, it may be one reason why the existing literature does not show a consistent benefit or inhibitory effects of serum on biofilm formation.

### 3.6. Effects of HI FBS on C. glabrata Biofilm Structure

The effects of HI FBS on biofilm structure were visualized using confocal microscopy. Images showed that *C. glabrata* formed a single layer of yeast cells on the glass surface with or without HI FBS. With the HI FBS coating, the *C. glabrata* biofilm exhibited a less dense structure (Figure 6A). In contrast, in the absence of HI FBS, *C. glabrata* formed a more densely populated biofilm with larger clusters of cells (Figure 6B).

Quantitative analysis of the confocal images using COMSTAT2 revealed a strong and consistent effect of heat-inactivated FBS on biofilm formation across all three biological replicates (Table 2). Substratum coverage was reduced from 47.1 +/− 7.9 in the absence of HI FBS to 3.8 +/− 0.4 under HI FBS conditions, a ~12-fold reduction (*p* = 0.01). Biovolume showed a parallel decrease from 1.96 +/− 0.62 to 0.16 +/− 0.07 µm^3^/µm^2^ (*p* = 0.04). The direction of effect was consistent across every biological replicate (no HI FBS > HI FBS for both coverage and biovolume). In contrast, the mean thicknesses of biofilm-positive regions were comparable between conditions (5.60 +/− 0.74 vs. 4.73 +/− 0.75 µm; *p* = 0.23), and the maximum biofilm thickness was nearly identical (9.35 +/− 0.18 vs 8.91 +/− 0.23 µm; *p* = 0.06), indicating that where biofilm formed in the presence of HI FBS, its vertical architecture was not affected. The roughness coefficient Ra* was also assessed using COMSTAT2, and slightly elevated roughness was observed under HI FBS conditions (1.83 +/− 0.10 vs. 1.11 +/− 0.15; *p* = 0.003). The surface-to-volume ratio was more than doubled (12.60 +/− 2.01 vs. 6.09 +/− 1.78 µm^−1^; *p* = 0.014) in the presence of HI FBS. Both of these observations were consistent with the sparse, patchy distribution of biofilm structures observed in the HI FBS condition relative to the more confluent biofilm in the absence of HI FBS.

The measured reductions in biofilm formation based on substratum coverage and biovolume in the presence of HI FBS as observed in the confocal images appear to correlate better with results from crystal violet staining than with those from XTT reduction, which suggests that HI FBS may have a positive influence on metabolic activity in mature biofilms or that the cells in 24 h biofilms are in different physiological states in the presence of HI FBS compared to controls in the absence of HI FBS. However, without the viable cell numbers in the biofilms, the exact effect of HI FBS on metabolic activity per attached cell cannot be ascertained.

## 4. Conclusions

This study aimed to evaluate the effects of fetal bovine serum on *C. glabrata* biofilm formation and initial attachment to surfaces commonly used in biofilm studies. Using both crystal violet staining and XTT reduction assays, the results showed significant reductions in biofilm formation and initial cell attachment in the presence of HI FBS. The reductions in biofilm formation due to HI FBS were observed on all surfaces tested (polystyrene, vinyl, and glass) and to different extents based on the type of surface. The results suggested that biofilm reduction due to FBS was likely not generated due to heat inactivation and that it reduced cell attachment rather than cell growth based on the absence of significant differences in maximum specific growth rates of planktonic cells with or without medium supplementation with FBS. However, we cannot rule out the possibility of reduced cell proliferation on the surface as a contributing factor. Interestingly, a larger effect was consistently observed in crystal violet staining compared with XTT data. Confocal imaging results appeared to correlate with the total biomass measured by crystal violet staining, but not so much with XTT reduction activities. Differences in what the two assays measure (crystal violet binds to cellular components and other biomolecules present in the extracellular polymeric substances, while the XTT assay measures the capacity of the cell to reduce this compound) likely play a part in the discrepancies observed between the two assays. However, given that significantly fewer cells were observed in the confocal imaging, but no corresponding decrease was measured in XTT reduction, it is possible that the presence of HI FBS causes changes in cellular physiology and metabolism that enhance cellular ability to reduce XTT. Since only one strain of *C. glabrata* was used in this study, future work in other isolates is needed to determine whether the same phenomenon is present in other isolates and *Candida* spp. Overall, our results showed that in *C. glabrata*, serum reduces biofilm formation and initial cell attachment but not the planktonic cell growth rate, and that combining multiple biofilm quantification techniques with imaging and growth kinetic analyses can provide complementary data on the effects of experimental factors on biofilm formation, while supporting further study of the serum components and cell–surface interaction mechanisms involved.

## Figures and Tables

**Figure 1:**
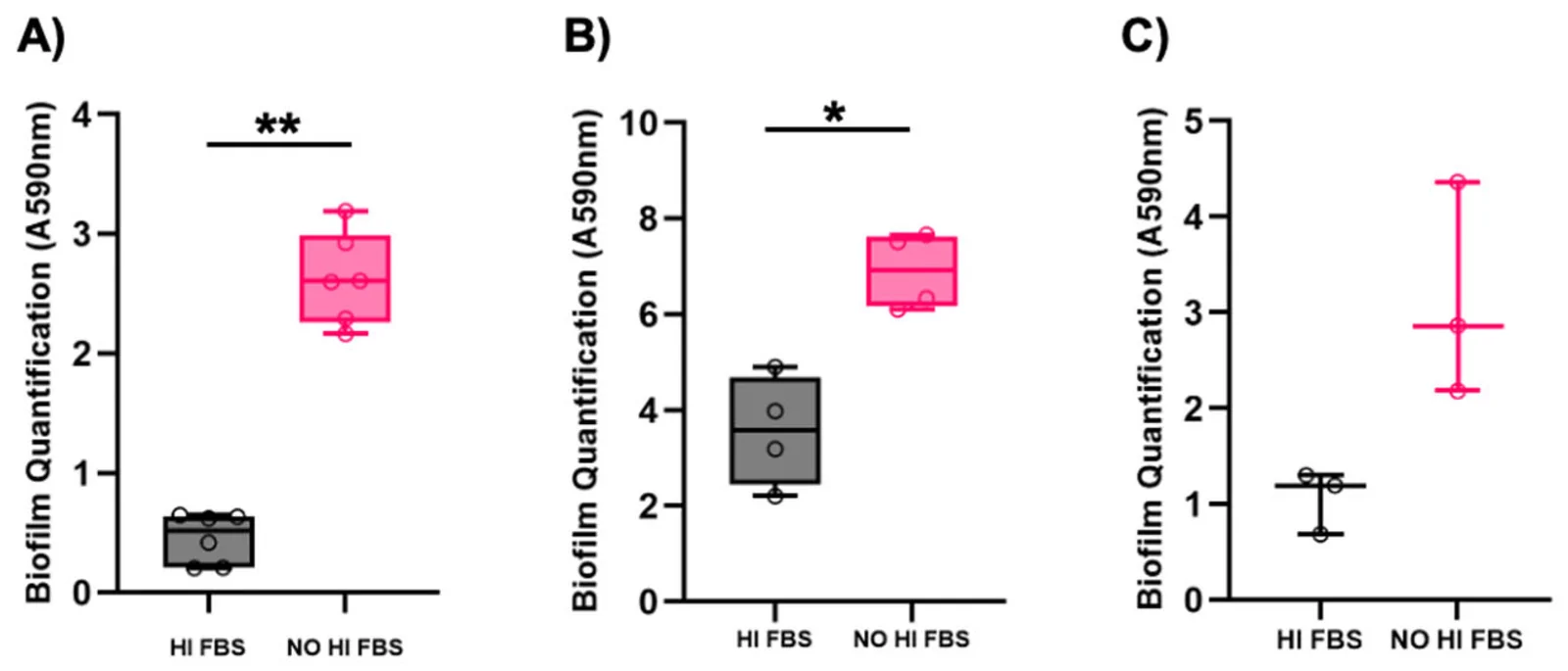
Effects of HI FBS on biofilm formation of *C. glabrata* on different surfaces. (**A**) Biofilm formation on half HI FBS pre-coated and half non-coated 96-well plates. Error bars are standard deviations from 6 biological replicates with 24 technical replicates per biological replicate. (**B**) Biofilm formation on HI FBS-coated and non-coated plastic coverslips. Error bars are standard deviations from 3 biological replicates, with 6 technical replicates per biological replicate. (**C**) Biofilm formation on HI FBS-coated and non-coated glass coverslips. Error bars are standard deviations. *: *p*-value < 0.05. **: *p*-values < 0.001.

**Figure 2:**
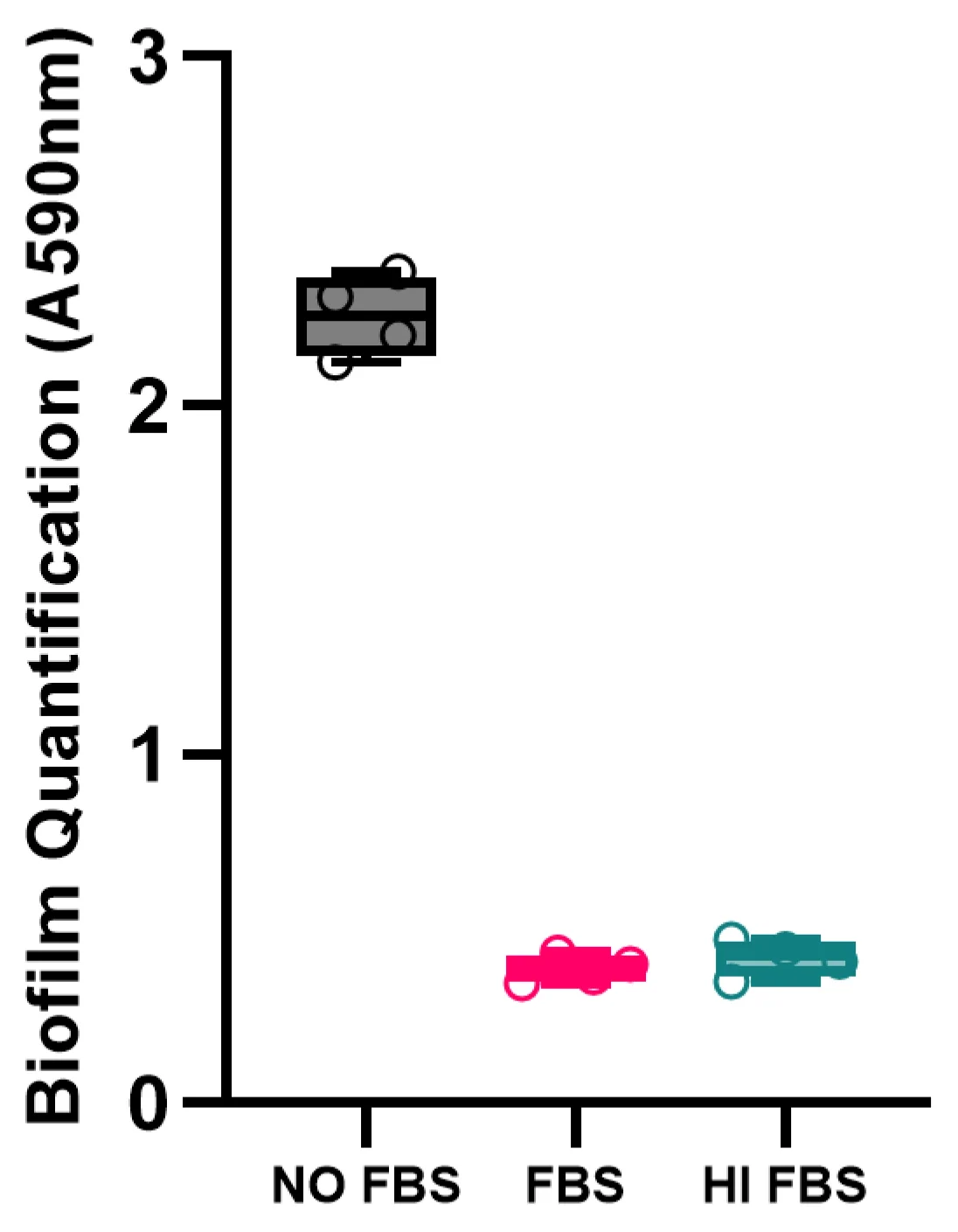
Effect of heat inactivation of FBS on *C. glabrata* biofilm formation. Error bars are standard deviations from three biological replicates, with twelve technical replicates per biological replicate.

**Figure 3:**
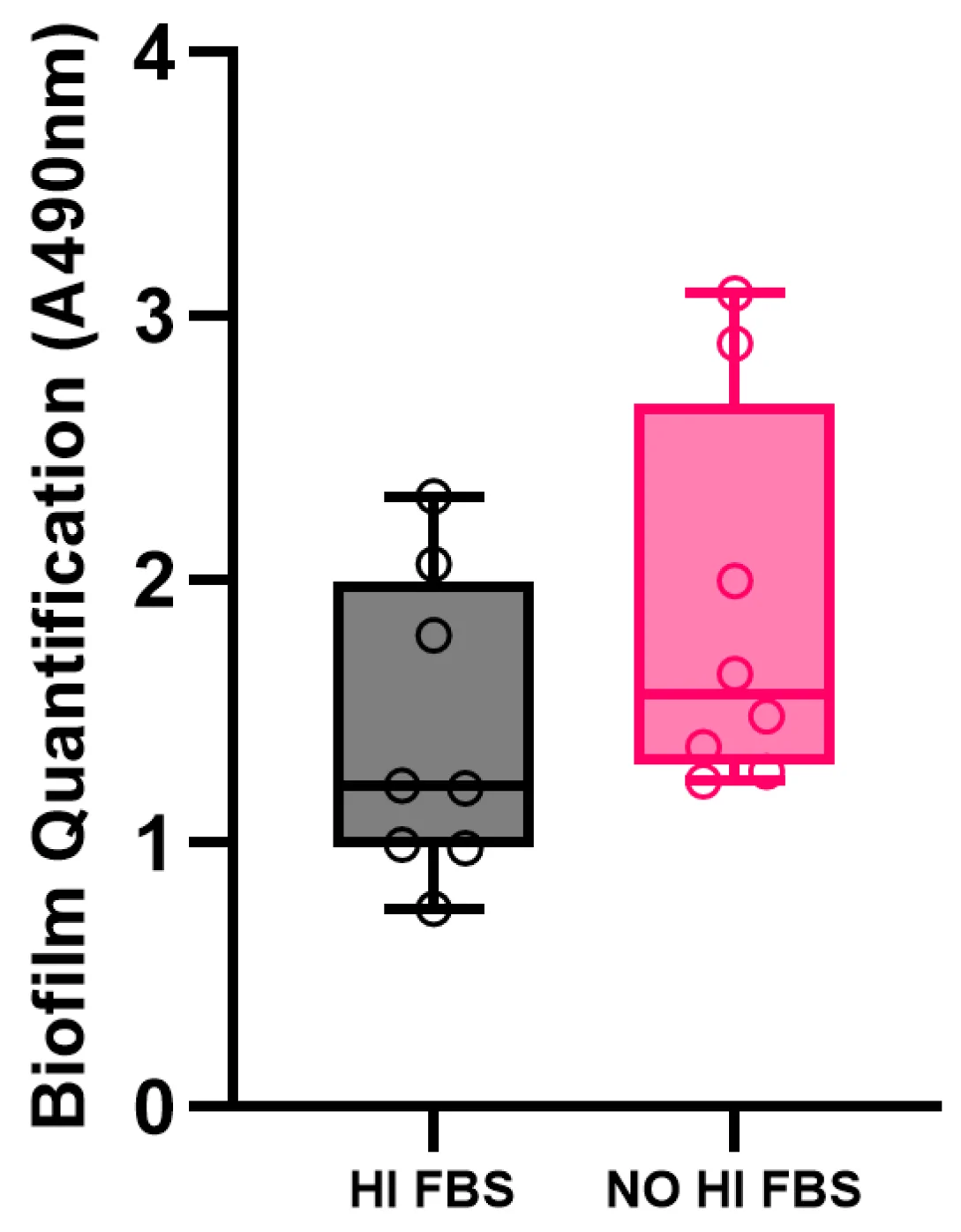
Biofilm metabolic activity based on XTT reduction assay between HI FBS-coated and non-coated wells after 24 h of incubation at 37 °C. Error bars are standard deviations from eight biological replicates, with twenty-four technical replicates per biological replicate.

**Figure 4:**
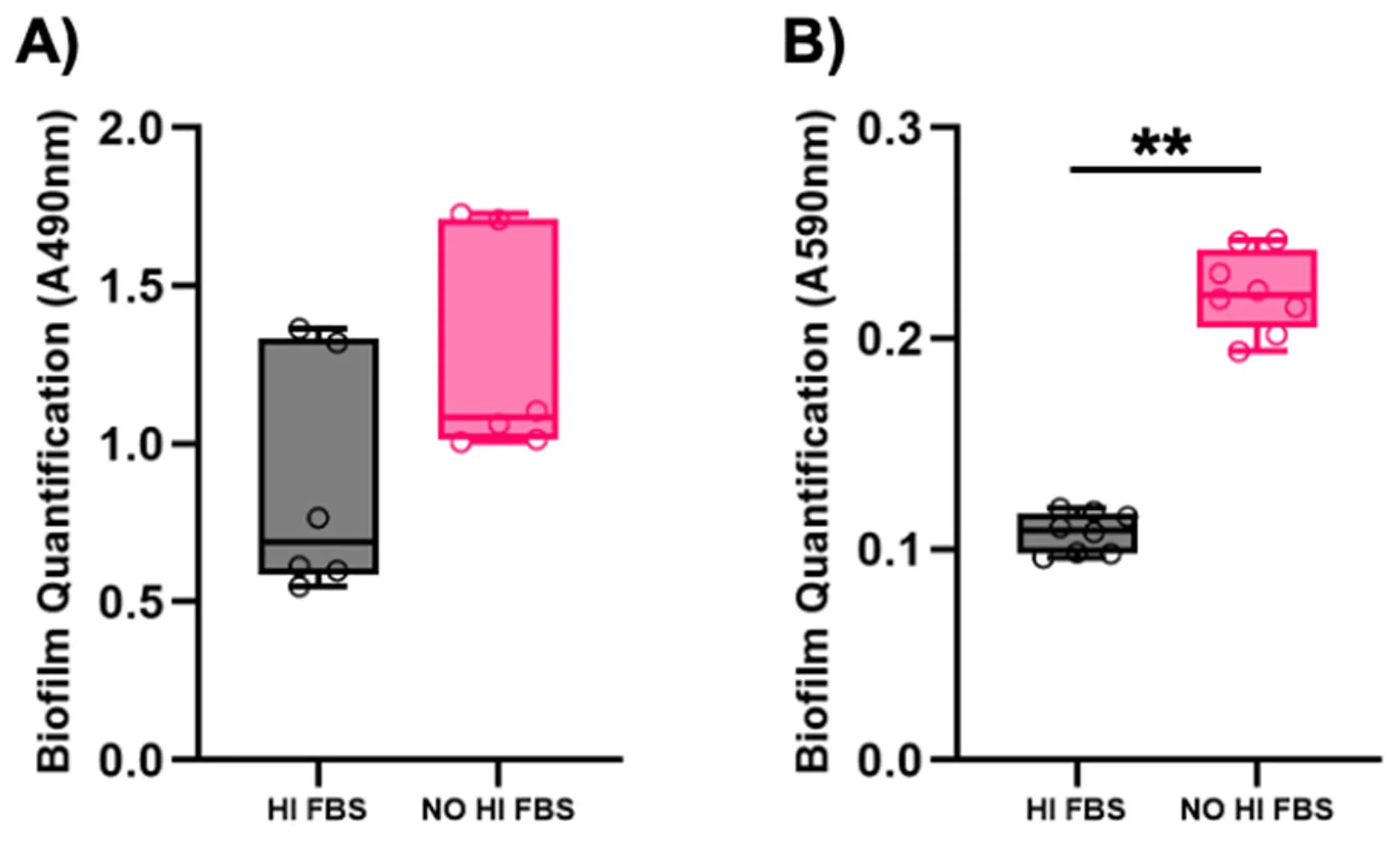
Effects of HI FBS on initial cell attachment measured using (**A**) XTT reduction assay and (**B**) crystal violet staining. Error bars are standard deviations from six biological replicates, with twenty-four technical replicates per biological replicate. **: *p*-values < 0.001.

**Figure 5:**
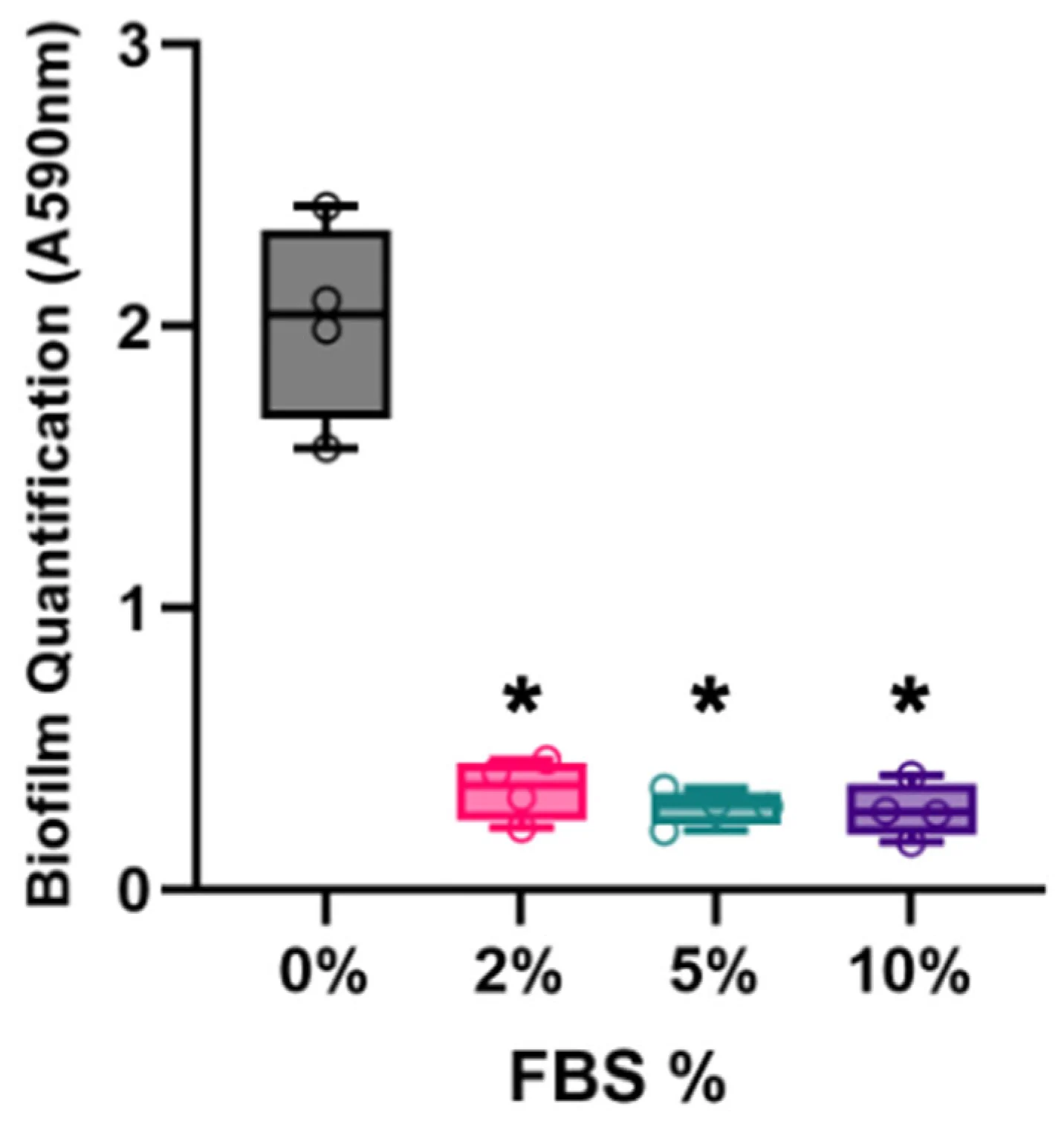
Effects of HI FBS supplementation in RPMI 1640 for biofilm formation. Error bars are standard deviations from four biological replicates, with six technical replicates per biological replicate. *: *p*-value < 0.05 compared with 0% FBS.

**Figure 6:**
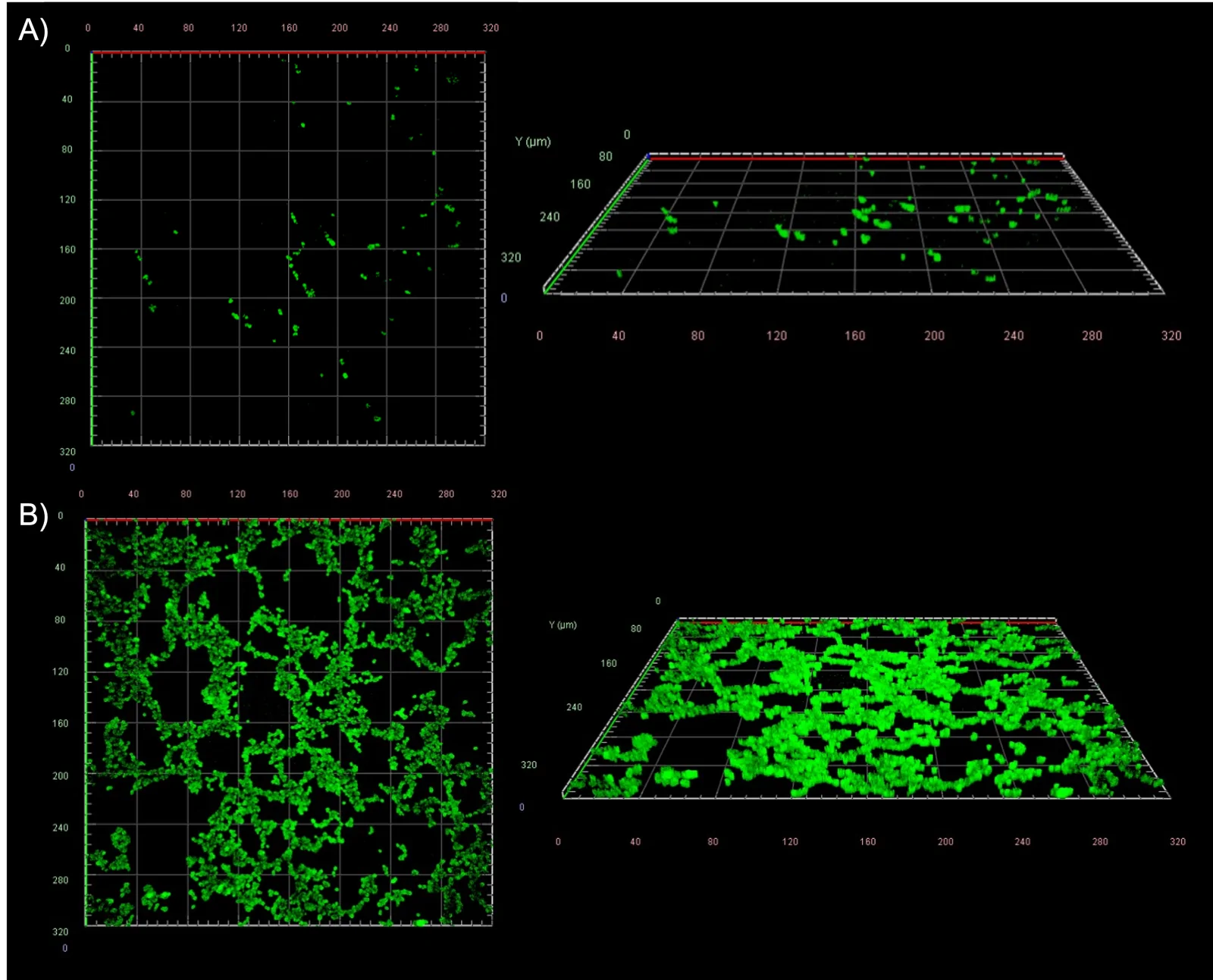
Confocal microscope images of *C. glabrata* (CgYFP). Filter: Fluorescein Isothiocyanate (FITC). (**A**) Magnification at 40× of biofilm formed on HI FBS-coated glass coverslip; ~10 µm thickness. (**B**) Magnification at 40× of biofilm formed on uncoated glass coverslip; ~10 µm thickness.

**Table 1:** Effects of HI FBS on specific growth rate and lag time on planktonic cell growth. Values are averages +/− standard deviations from six biological replicates, with six technical replicates per biological replicate. Bolded and italicized values have *p*-values < 0.05 from 0% FBS.

FBS Concentration	µ (per h)	Lag Time (h)
0%	0.27 ± 0.03	1.63 ± 0.21
2%	0.29 ± 0.02	1.42 ± 0.21
5%	0.27 ± 0.01	***1.25 ± 0.28***
10%	0.27 ± 0.01	***1.01 ± 0.35***

**Table 2:** COMSTAT2 analysis of confocal images.

Parameter	NO HI FBS (Mean ± SD)	HI FBS (Mean ± SD)	Ratio (HI FBS/NO HI FBS)	*p*-Values
Coverage	47.106 ± 7.906	3.820 ± 0.362	0.08	0.011
Biovolume	1.958 ± 0.619	0.161 ± 0.067	0.08	0.036
Mean biomass thickness	5.600 ± 0.745	4.728 ± 0.749	0.84	0.226
Max thickness	9.352 ± 0.179	8.907 ± 0.225	0.95	0.058
Ra*	1.109 ± 0.146	1.833 ± 0.096	1.65	0.003
S/V	6.087 ± 1.784	12.604 ± 2.009	2.07	0.014

## Data Availability

The original contributions presented in this study are included in the article/Supplementary Materials. Further inquiries can be directed to the corresponding author.

## Supplementary Materials

The following supporting information can be downloaded at https://www.mdpi.com/article/10.3390/microorganisms14102125/s1, Figure S1. Effects of HI FBS coating time on *Candida glabrata* biofilm formation. Error bars are standard deviations from four biological replicates, with six technical replicates per biological replicate. Figure S2. Confocal images of additional biological replicates of *C. glabrata* biofilms on (A) HI FBS-coated coverslips and (B) non-coated coverslips.

## Abbreviations

The following abbreviations are used in this manuscript:

HI FBS	Heat-inactivated fetal bovine serum
XTT	2,3-Bis-(2-Methoxy-4-Nitro-5-Sulfophenyl)-2H-Tetrazolium-5-Carboxanilide Tetrazolium Salt
