# Supplementary material for: Effects of Fetal Bovine Serum on Biofilm Formation and Cell Attachment of Candida glabrata
Source: Microorganisms. 2026 Sep 22;14(10):2125. doi: 10.3390/microorganisms14102125 (PMC13707765; doi:10.3390/microorganisms14102125)
Supplement: Supplementary file 1 [file microorganisms-14-02125-s001.zip › microorganisms-4513614-supplementary.pdf]

microorganisms

Supplemental Figure S1. Effects of HI FBS coating time on *Candida glabrata* biofilm formation. Error bars are standard deviations from four biological replicates with six technical replicates per biological replicate.

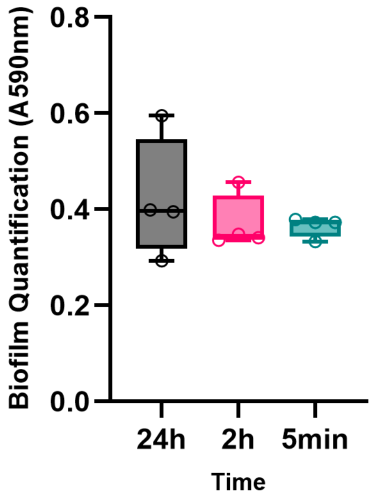

Supplemental Figure S2. Confocal images of additional biological replicates of *C. glabrata* biofilms on A) HI FBS coated coverslips and B) non-coated coverslips.

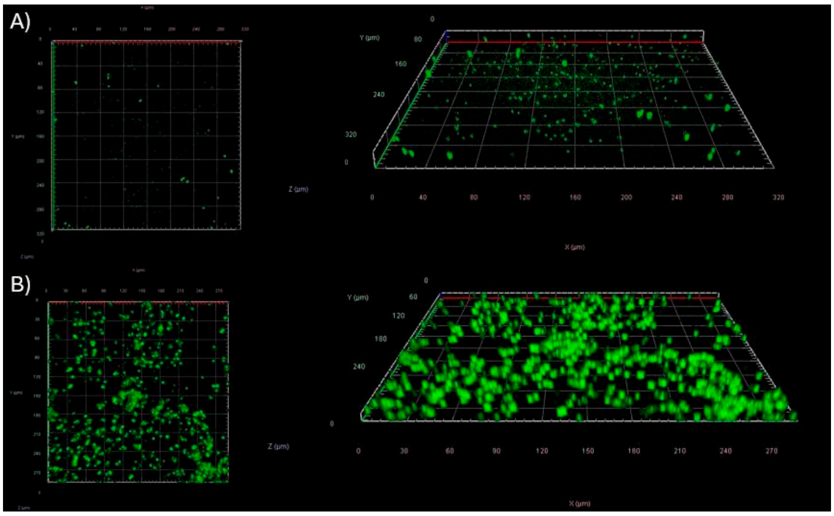

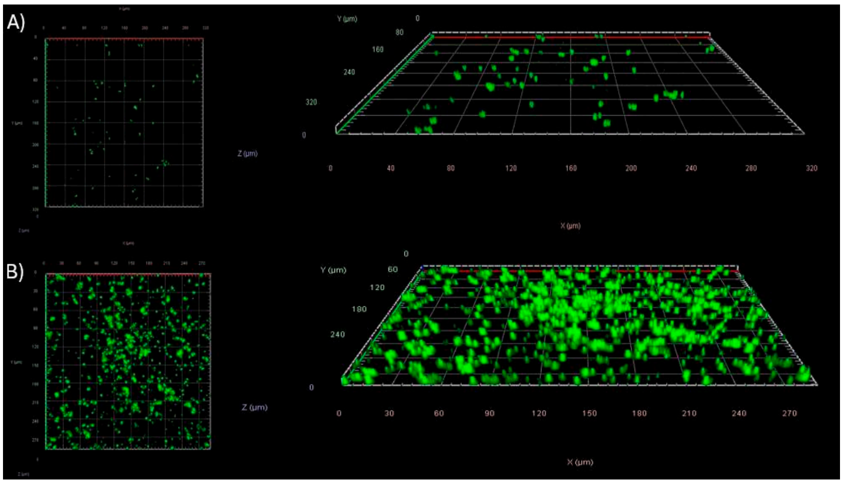
A)
B)
Y (µm)
Z (µm)
X (µm)
0
80
160
240
320
0
40
80
120
160
200
240
280
320
0
60
120
180
240
0
30
60
90
120
150
180
210
240
270

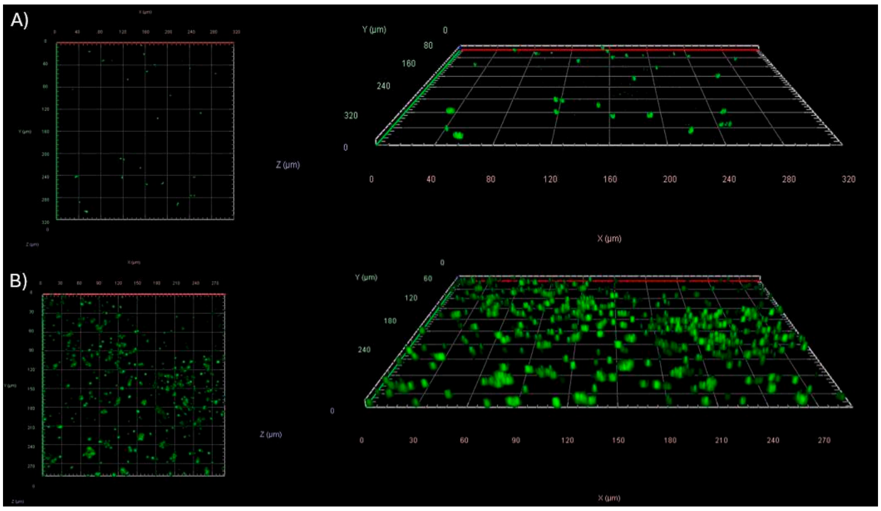
A)
B)
Y (µm)
Z (µm)
X (µm)
0
80
160
240
320
0
40
80
120
160
200
240
280
320
0
60
120
180
240
0
30
60
90
120
150
180
210
240
270
